# The nature and activity of liaison mental services in acute hospital settings: a multi-site cross sectional study

**DOI:** 10.1186/s12913-020-05165-x

**Published:** 2020-04-15

**Authors:** Sonia Saraiva, Elspeth Guthrie, Andrew Walker, Peter Trigwell, Robert West, Farag Shuweidi, Mike Crawford, Matt Fossey, Jenny Hewison, Carolyn Czoski Murray, Claire Hulme, Allan House

**Affiliations:** 1 Leeds Institute of Health Sciences, University of Leeds, Leeds, UK; 2 Clinical Research Network National Coordinating Centre, National Institute of Health Research Clinical Research Network, Leeds, UK; 3 National Inpatient Centre for Psychological Medicine, Leeds and York Partnership NHS Foundation Trust, Leeds, UK; 4 College Centre for Quality Improvement, Royal College of Psychiatrists, London, UK; 5 Department of Brain Sciences, Faculty of Medicine, Imperial College, London, UK; 6 Veterans and Families Institute for Military Research, Faculty of Health, Social Care and Education, Anglia Ruskin University, Chelmsford, UK; 7 College of Medicine and Health, University of Exeter, Exeter, UK

**Keywords:** Liaison psychiatry, Mental health liaison services, Activity survey

## Abstract

**Background:**

To describe the clinical activity patterns and nature of interventions of hospital-based liaison psychiatry services in England.

**Methods:**

Multi-site, cross-sectional survey. 18 acute hospitals across England with a liaison psychiatry service. All liaison staff members, at each hospital site, recorded data on each patient they had face to face contact with, over a 7 day period. Data included location of referral, source of referral, main clinical problem, type of liaison intervention employed, staff professional group and grade, referral onto other services, and standard assessment measures.

**Results:**

A total of 1475 face to face contacts from 18 hospitals were included in the analysis, of which approximately half were follow-up reviews. There was considerable variation across sites, related to the volume of Emergency Department (ED) attendances, number of hospital admissions, and work hours of the team but not to the size of the hospital (number of beds). The most common clinical problems were co-morbid physical and psychiatric symptoms, self-harm and cognitive impairment. The main types of intervention delivered were diagnosis/formulation, risk management and advice. There were differences in the type of clinical problems seen by the services between EDs and wards, and also differences between the work conducted by doctors and nurses. Almost half of the contacts were for continuing care, rather than assessment. Eight per cent of all referrals were offered follow up with the LP team, and approximately 37% were referred to community or other services.

**Conclusions:**

The activity of LP services is related to the flow of patients through an acute hospital. In addition to initial assessments, services provide a wide range of differing interventions, with nurses and doctors carrying out distinctly different roles within the team. The results show the volume and diversity of LP work. While much clinical contact is acute and confined to the inpatient episode, the LP service is not defined solely by an assessment and discharge function; cases are often complex and nearly half were referred for follow up including liaison team follow up.

## Background

Liaison psychiatry services in acute hospitals are now reasonably well established because of a widespread recognition of the morbidity associated with mental health problems in that setting [1]. For example, a recent systematic review of mental health attendances to ED reported that mental health disorders account for 4% of ED attendances, [2]. The largest study in this field is the European Consultation-Liaison Workgroup (ECLW) Collaborative Study, which collected data from 56 different liaison services in 11 European Countries and included 10,560 patients [3]. The mean consultation rate in the ECLW study was 1.4% of all admissions. Estimated rates of mental health consultations varied from 0.7 to 5% [4–9].

Recent national surveys in the UK have found that all 179 acute general hospitals large enough to have their own ED are served by a liaison psychiatry service. However, service structure and size varies considerably. There is now underway an expansion in liaison psychiatry services in England [10] with a government target that at least 50% of all liaison psychiatry services in acute hospitals should be adequately staffed (according to commissioning guidelines) by 2020/21, 70% by 2023/24 and 100% in the long term [11]. Lying behind this expansion is a hope that these more extensive services will bring about a reduction in costs, largely through reduced length of hospital stay for people with physical and mental health problems. For this reason, most recent evaluations have focused primarily on organisational outcomes such as length of hospital stay or response times.

More widely, there is evidence of a range of other positive outcomes of liaison psychiatry services, including better access to mental healthcare for patients in acute settings and patient and referrer satisfaction [12–15]. However only a small number of evaluations providing data on the nature of clinical work [16], by whom it was provided and any evidence of clinical outcome [17]. The evidence base is small and mainly based upon local service evaluation, and further larger-scale evaluations are required [12, 18].

There are three challenges inherent in evaluating liaison psychiatry services: variability in what LP services actually provide in terms of clinical care; variability in structures, and uncertainty about the clinical and service-level outcomes that should be collected routinely.

Regarding the range of interventions delivered by LP services, the number of patients they see, and the clinical outcomes they achieve - the most common reasons for referral to liaison services in the ELCW study were current psychiatric symptoms (47%), self-harm (17%), unexplained physical complaints (22%) and substance misuse (10%). Type of liaison intervention and clinical outcome was not recorded. Several recent local evaluations of LP services have provided estimates of activity for specific hospitals in different parts of England. However, it is difficult to generalise from these local evaluations to a wider national picture due to the wide variations between services.

There is considerable variation in the size and composition of LP services and the different sizes of hospitals they serve [13, 17, 19–21]. Most liaison services are multidisciplinary and many ED liaison psychiatry services are nurse-led (supported by psychiatrists via on-call systems) but it is unclear whether nurses and doctors perform similar roles in a liaison setting, or whether both are necessary for a comprehensive LP service.

In previous work, we have shown that liaison services can be grouped into four patterns of service delivery depending upon size of service, salience of acute work, provision of outpatient clinics and differentiation of an age- specific component of the service [22]. These comprise briefly: small services that operate weekly on a 9 a.m. to 5 p.m. basis; services that provide 24/7 acute work and comparatively little non-acute work; services that provide acute and non-acute work; and services that are less focused on the acute care pathway with separate teams for the care of adults of working age and older adults. It is unclear whether these different configurations of liaison service see different kinds of patients and have different levels of activity (number of face-to-face patient contacts).

In relation to outcomes, the Royal College of Psychiatrists, Liaison Faculty has recently developed a framework (FROM-LP) for the clinical evaluation of LP services [23]. This includes recommendations to use a simple set of measures to assess outcome, and a typology to describe different forms of clinical interventions employed by LP services; the-Identify and Rate the Aim of the Contact (IRAC) scale [23]. IRAC has shown good clinical utility in correctly identifying the type of interventions implemented by LP teams [24]. The Royal College of Psychiatrists recommends that all LP services use the framework to improve the recording of clinical data, with a caveat that there are inherent problems in assessing clinical outcomes in LP services due to contact with most patients being a single, brief, consultation for complex, persistent problems. Three studies have used similar frameworks to FROM-LP to describe the type of interventions delivered by LPs [16, 21, 24]. However, it remains unclear whether such measures are used routinely by many services, and if so for which clinical or organisational outcomes.

The aim of this study was to determine the nature and activity of work carried out by LP services across England, to enable us to develop a framework for evaluating the effectiveness and cost-effectiveness of selected liaison services. This work formed part of the second phase of a programme of research funded through the National Institute for Health Research Health Service Health Services and Delivery Research Programme to evaluate the cost-effectiveness and efficiency of different configurations of liaison psychiatry services in England (Measurement And Evaluation of Service Types, Referral patterns and Outcomes LP-MAESTRO 13/58/08. NIHR HS & DR In progress). The specific objectives were to; a) provide estimates of the activity of LP services (number of face to face patient contacts); b) determine the frequency of different types of psychosocial interventions provided by LP services c) compare workload from ED and hospital wards d) compare the type of work undertaken by doctors and nurses within the LP services e) compare the workload and type of work undertaken by different types of LP services according to our four categories of service delivery.

## Methods

NHS Research Ethics (REC reference: [15] /NS/0025) and NHS Trust level approvals were obtained. We have followed the ‘Strengthening the Reporting of Observational Studies in Epidemiology’ guidelines for reporting of observational studies [25].

All 168 hospitals in England with an ED and a LP service in 2015 were identified, and all participated in a survey about staffing and service configuration, which has been reported elsewhere [22]. Twenty three of the 168 (13.7%) services agreed in principal to participate in a more detailed activity survey, of which 19 were practically able to participate. After initial data analysis one hospital was excluded because of incomplete data, therefore 18 hospitals were included in the final analysis reported here, including at minimum two services from each of the four liaison categories described above.

LP services were required to record all face-to-face clinical contact with patients over seven consecutive days in March or June 2017, dependent upon local feasibility. Standardised paper survey forms were provided to all the LP services. At each site, a senior member of the LP service took responsibility for distributing the forms and co-ordinating responses. All LP service staff who had face-to-face clinical contact with patients during the survey week were required to record the interaction: each member of staff completed one form per shift. If a patient was seen by more than one member of staff during the study period, staff were asked to note this in a section on comments but not to complete it as a separate contact, so as to avoid double counting.

We estimated the number of hospital admissions and ED attendances per week by dividing annual rates derived from Hospital Episode Statistics (HES) [26] for the period 01 April 2016 to 31 March 2017 by 52. We then used these estimates as denominators to calculate an estimated proportion of patients seen by LP services, both on the wards and in ED. As HES data are published according to NHS Trust rather than individual hospitals it was not always possible to extract specific data for every surveyed hospital. When data were aggregated with other hospitals (when an NHS Trust had more than one acute hospital), we requested this information directly from the acute hospital in question. Data were available for 14 hospitals regarding admissions and 17 hospitals regarding ED attendances.

The study was a cross-sectional multi-site observational study. To support LP staff in completing the survey while minimising the potential for missing data, the survey reporting forms were designed to be both quick and easy to complete and a completed example was provided for guidance. The following information was collected: background and setting (e.g. ED, ward) of the person who referred to the LP team; health profession of LP team member who had contact with the patient; reason for referral (problems of adjustment to illness; medically unexplained symptoms; overt psychiatric/psychological symptoms in the presence of physical illness; cognitive impairment; self-harm; acute behavioural disturbance; alcohol or substance misuse; and/or other problem), first or repeated contact; type of liaison intervention was determined using pre-defined categories [16] (assessment diagnosis and formulation; providing advice/signposting; management of risk; Mental Health Act Assessment or assessment of capacity; medication management; management of disturbed behaviour; other kind of intervention); use of standardised assessment measures; and whether the person was referred on to other services.

Continuous data are presented as means and standard deviations for comparing normally distributed data, and, as medians and inter-quartile ranges for comparing not normally distributed data. Categorical data are presented as number and percentages. Comparisons were made for continuous and categorical data using parametric or non-parametric tests depending whether the data were normally or not normally distributed.

Multilevel binary logistic regression was performed [27] to estimate the relationship between the type of clinical problem seen and the type of liaison intervention delivered for two dependent variables; staff type (either doctors or nurses) and location of work (either ward work or ED). As patients were nested in hospitals, the hospitals were grouped into 4 clusters [22], according to the type of liaison service, which was operating at each of the hospitals, and these 4 clusters were entered as predictors into the analyses. Results are presented in the form of odds ratios (OR) and 95% Confidence Intervals (CIs). To test the significance of any effects regarding type of liaison service, we ran a likelihood ratio test comparing the null multilevel model with a null single-level model.

We did not know the intra-class correlation coefficient (ICC) for our primary outcome prior to analysis, but we did anticipate it to be in the region of 0.01 to 0.05. To be conservative, we took ICC = 0.05. This gives a design effect of 74.7. Using this value there was 90% power with an effective sample size of *n* = 1194, after accounting for clustering within centre [28–30].

## Results

Characteristics of the hospital sites and the activity reported by the liaison teams are shown in Table 1. The hospitals varied in size, number of ED attendances and locality across England, with ten hospitals from the North of England and eight from the South. There was considerable variation in the staffing levels of the individual liaison services and their reported activity. Five LP services provided 24/7 services, and these services reported higher levels of overall activity than non 24/7 services (median = 113, IQR = 66.5–171.5 vs median = 55, IQR = 39.5–108; U = 12.5, *p* = 0.05) and a higher number of LP staff (median = 19, IQR = 15.5–27 vs median = 8, IQR = 4.6–19.5; U = 12, *p* = 0.04).

**Table 1:** Activity at the 18 hospital sites according to admissions per week, ED attendances and LPS activity

*Beds (n)*	*24 h*	*LP staff completing activity recording(n)*	*Admissions per week (annual figure /52)*	*ED attendances per week (annual figure/52)*	*LP activity per week*	*LP activity from ED % estimated activity*	*LP activity from Ward % estimated activity*	*LP activity from other places*
1128	Yes	22	2758	2185	113	55 (2.5%)	49 (1.8%)	9
781	Yes	32	2381	2165	172	27 (1.2%)	124 (5.2%)	21
488	No	10	1198	1594	46	12 (0.8%)	31 (2.6%)	3
448	No	18			109	23	84	2
307	No	6	993	729	27	5 (0.7%)	22 (2.2%)	0
700	Yes	19		3766	171	62 (1.6%)	96	13
988	No	21	3113	2097	129	39 (1.9%)	44 (1.4%)	46
525	No	8	1763	1297	41	23 (1.8%)	13 (0.7%)	5
1113	No	22	2319	1701	107	58 (3.4%)	31 (1.3%)	18
450	Yes	18	1126	1647	78	36 (2.2%)	35 (3.1%)	7
1477	No	13	1994	2718	63	17 (0.6%)	35 (1.8%)	11
696	No	8	2089	1713	38	12 (0.7%)	22 (1.1%)	4
557	No	1	1302	1401	55	31 (2.2%)	19 (1.5%)	5
1943	No	22		2652	151	26 (1.0%)	95	30
439	No	3	1516	1011	42	5 (0.5%)	37 (2.4%)	0
227	No	5	705	817	58	15 (1.8%)	42 (6.0%)	1
719	Yes	13		1356	55	25 (1.8%)	18	12
222	No	4	935	897	20	8 (0.9%)	11 (1.2%)	1

There was a positive correlation between the number of patients seen by the liaison teams and the number of ED attendances per week (*r* = 0.78, *p* = 0.001) and the number of hospital admissions (r = 0.72, *p* = 0.004). The estimated ward consultation rate (number of referrals divided by average weekly admissions) ranged from 0.7 to 6.0% (mean 2.2%) for the 14 hospitals for which we had hospital admission data. The estimated ED consultation rate (number of referrals divided by average weekly attendance rate) was lower and ranged from 0.5 to 3.4% (mean 1.5%) for the 17 hospitals for which we had ED attendance data.

After cleaning data, 1475 patient contacts from 18 sites were included in the analysis. Referrals not containing data on face-to-face contacts were excluded (*n* = 204). We recognise the work of LP services goes beyond direct assessment (e.g. telephone contacts, team meetings, case management meetings, administrative work, liaison with other health professionals, educational support), but non-direct contacts were beyond the scope of this research.

Most contacts were ward-based (55%, *n* = 808), with one third in ED (33%, *n* = 479) and the rest involving a variety of other services (13%, *n* = 188). The main sources of referral were doctors (48%, *n* = 700) and nurses (38%, *n* = 561) from the same acute hospital as the LP service. Other referrals came from LP colleagues (7%, *n* = 104), services outside the hospital (4%, *n* = 59) and other services (which were unspecified) (3.5%, *n* = 51). Just over half the contacts were initial assessments (53%, *n* = 779) and the remaining were follow-up assessments (47%, *n* = 694). The majority of patients were seen by either a mental health nurse (62%, *n* = 914) or a doctor (29%, 435) with a relatively small percentage seen by other mental health professionals -including social workers, psychologists and occupational therapists (8%, *n* = 126).

The main clinical problems and type of intervention are shown in Table 2. More than one clinical problem or type of intervention could be recorded for each patient. The most common reasons for referral to LP services were psychiatric symptoms (36%, *n* = 640) that is - patients with co-morbid physical and mental health problems - self-harm (23%, *n* = 417) and cognitive impairment (16%, *n* = 281). Other clinical problems (including alcohol and drug related problems, behavioural disturbance, medically unexplained symptoms, problems with psychological adjustment to illness, and ‘other’) were individually much less frequent but collectively accounted for over one quarter of all contacts (26%, *n* = 467).

**Table 2:** Main clinical problem area seen and type of intervention for LPS at 18 acute hospitals according to LP professional background and location

	Responses	LP professional background		Location of referral	
	*n* (%)	Dr *n* (%)	Nurse *n* (%)	ED *n* (%)	Ward *n* (%)
Main clinical problem					
Adjustment to illness	89 (4.9)	24 (4.4)	42 (3.8)	4 (0.7)	61 (6.1)
Medical unexplained symptoms	53 (2.9)	25 (4.6)	22 (2)	4 (0.7)	26 (2.6)
Psychiatric symptoms	640 (35.5)	198 (36.1)	385 (35)	225 (38.8)	343 (34.2)
Cognitive impairment	281 (15.6)	120 (21.9)	140 (12.7)	8 (1.4)	266 (26.5)
Self-harm	417 (23.1)	90 (16.4)	303 (27.5)	219 (37.8)	140 (14)
Acute behaviour disturbance	111 (6.1)	55 (10)	49 (4.5)	26 (4.5)	79 (7.9)
Alcohol and/or drugs	150 (8.3)	29 (5.3)	115 (10.4)	83 (14.3)	54 (5.4)
Other	64 (3.5)	8 (1.5)	45 (4.1)	11 (1.9)	34 (3.4)
Total	1805 (100)	549 (100)	1101 (100)	580 (100)	1003 (100)
Type of Intervention					
Assessment and diagnosis formulation	750 (32.9)	222 (30.5)	459 (33.4)	257 (34.1)	428 (34.6)
Providing advice/signposting	512 (22.5)	132 (18.2)	344 (25)	191 (25.4)	259 (20.9)
Management of risk	504 (22.1)	134 (18.4)	352 (25.6)	242 (32.1)	189 (15.3)
Assessment of mental capacity/MHA	94 (4.1)	59 (8.1)	33 (2.4)	25 (3.3)	58 (4.7)
Medication management	160 (7.0)	109 (15)	46 (3.3)	6 (0.8)	125 (10.1)
Management of disturbed behaviour	123 (5.4)	57 (7.8)	58 (4.2)	16 (2.1)	100 (8.1)
Other	135 (5.9)	14 (1.9)	83 (6)	16 (2.1)	79 (6.4)
Total	2278 (100)	727 (100)	1375 (100)	753 (100)	1238 (100)

The most frequent type of interventions employed by the liaison teams were: assessment, diagnosis and formulation (33%, *n* = 750); provision of guidance and advice (23%, *n* = 512); and risk management (22%, *n* = 501). Other types of liaison interventions (Mental Health Act work or assessment of capacity, management of acute behavioural disturbance, medication management and ‘other interventions’) were less frequent but accounted for more than one in five (22%, *n* = 512) of all the interventions delivered by the teams.

Following contact with liaison services, just under half of the patients were referred for further treatment (47%, *n* = 807, most commonly to community mental health teams (14%, *n* = 211). Follow up by the liaison team was arranged for 127 (8.5%) with smaller numbers of patients referred to: alcohol and drugs services (4%, *n* = 65); Improving Access to Psychological Treatment Services (3%, *n* = 43), General Psychiatry Single Point of Access (2%, *n* = 33), inpatient psychiatric services (1.6% *n* = 24); third sector services (1.5% *n* = 22) and psychology services (1.2%, *n* = 18). A further 165 patients (11%, *n* = 165) were referred to ‘other’ services, with no additional information provided.

Standardised measures were employed infrequently, with over two thirds of patients not completing any kind of standardised measure (70%, *n* = 984). FROM-LP, in its entirety, was reported as being used in only 15 patient contacts (1%): however, the Clinical Global Improvement Scale [31], the main outcome measure recommended by FROM-LP, was used in 100 patient contacts (7%). Other standardised measures used included the Mini Mental State Examination (*n* = 59, 4%) [32], Hospital Anxiety and Depression Scale (*n* = 19 1%) [33], Health of the Nation Outcome Scales (*n* = 24 2%) [34], CORE (*n* = 10, 1%) [35], and other measures which were not specified (*n* = 179, 13%).

There were differences between doctors and nurses in terms of the clinical problems they reported, the work they carried out, and whether the patient contact was on a ward or in the ED. The numbers and percentages of patients for each clinical problem and type of intervention are shown in Table 2 according to professional background (nurses and doctors).

Table 3 shows the results of a multilevel logistic regression analysis with ‘nurse or doctor’ as the dependent variable. A ratio above 1 indicates the likelihood of a nurse as opposed to a doctor carrying out a face to face contact with a patient with a specific clinical problem or providing an intervention. Nurses were less likely than doctors to see patients with medically unexplained symptoms (OR = 0.3, 95%CI: 0.1, 0.7), psychiatric symptoms (OR = 0.5, 95%CI: 0.4, 0.8) and acute behavioural disturbance (OR = 0.3, 95%CI: 0.2, 0.6). Nurses were also much less likely than doctors to carry out Mental Health Act work or capacity assessments (OR = 0.2, 95%CI: 0.1, 0.3) and medication management (OR = 0.2, 95%CI: 0.1, 0.3). Nurses were over seven times more likely than doctors to see patients in the ED setting (OR = 7.5, 95%CI: 4.2, 13.4), although there was no such difference in ward work. We did not examine any differences in the work of other health professionals, as the numbers were too small to make any meaningful comparisons.

**Table 3:** Multilevel logistic regression for LP professional background; nurses versus doctors

*Variables*	*OR*	*(95% CI)*	*p-value*
Intercept	3.6	(1.1–11.6)	0.03
Main clinical problem for contact			
Other (reference)	1.0		
Adjustment to illness	0.7	(0.4–1.4)	0.33
Medical unexplained symptoms	0.3	(0.1–0.7)	<.001
Psychiatric symptoms	0.5	(0.4–0.8)	<.001
Cognitive impairment	0.6	(0.4–1.0)	0.07
Self-harm	1.2	(0.7–1.9)	0.52
Acute behaviour disturbance	0.3	(0.2–0.6)	<.001
Alcohol and/or drugs	1.6	(0.9–2.7)	0.13
Type of Intervention			
Other (reference)	1.0		
Assessment and diagnosis formulation	0.9	(0.7–1.3)	0.75
Providing advice/signposting	1.2	(0.8–1.7)	0.34
Management of risk	0.8	(0.5–1.1)	0.17
Assessment of mental capacity/MHA	0.2	(0.1–0.3)	<.001
Medication management	0.2	(0.1–0.3)	<.001
Management of disturbed behaviour	0.9	(0.5–1.5)	0.59
Location of referral			
Other (reference)	1.0		
ED	7.5	(4.2–13.4)	<.001
Ward	1.1	(0.7–1.8)	0.61

Table 4 shows the results of a multilevel logistic regression comparing clinical problem and type of liaison intervention according to whether the contact was in ED or on a ward. A ratio above 1 indicates that the patient was more likely to be seen on the wards rather than ED.

**Table 4:** Multilevel logistic regression for location of referral: ward versus ED

*Variables*	*OR*	*(95% CI)*	*p-value*
Intercept	2.8	(1.3–6.0)	0.01
Main clinical problem for contact			
Other (reference)	1.0		
Adjustment to illness	14.4	(4.8–43.6)	<.001
Medical unexplained symptoms	5.4	(1.6–17.8)	0.01
Psychiatric symptoms	1.1	(0.8–1.7)	0.58
Cognitive impairment	23.0	(10.3–51.0)	<.001
Self-harm	0.5	(0.3–0.7)	<.001
Acute behaviour disturbance	1.1	(0.6–2.1)	0.75
Alcohol and/or drugs	0.5	(0.3–0.7)	0.00
Type of intervention			
Other (reference)	1.0		
Assessment and diagnosis formulation	0.6	(0.5–0.9)	0.01
Providing advice/signposting	0.8	(0.5–1.0)	0.09
Management of risk	0.6	(0.4–0.9)	0.01
Assessment of mental capacity/MHA	1.2	(0.6–2.1)	0.63
Medication management	13.6	(5.5–33.7)	<.001
Management of disturbed behaviour	2.5	(1.2–5.3)	0.01
Source of referral			
Other (reference)			
Referral from LP colleague	0.8	(0.3–1.9)	0.58
Referral from Dr. of same acute hospital	0.8	(0.4–1.5)	0.51
Referral from nurse of same acute hospital	0.5	(0.2–0.9)	0.02

There were considerable differences in the type of clinical problem LP services attended to in the ED or on the wards. Patients with psychological adjustment to physical illness (OR = 14.4, 95%CI: 4.8, 43.6), cognitive impairment (OR: 23.0, 95%CI: 10.3, 51.0) or medically unexplained symptoms (OR = 5.4, 95%CI: 1.6, 17.8) were much more likely to be seen in a ward setting, whereas patients with problems with alcohol/drugs (OR = 0.5, 95%CI: 0.3, 0.7) or self-harm (OR = 0.5, 95%CI: 0.3, 0.7) were much more common in the ED.

Liaison interventions also differed between the two settings. Liaison staff were much more likely to be involved in medication management (OR = 13.6, 95%CI: 5.5, 33.7) and assessment/management of disturbed behaviour (OR = 2.5, 95%CI: 1.2, 5.3) in a ward setting, whereas interventions involving assessment/diagnosis (OR = 0.6, 95%CI: 0.5, 0.9) and management of risk (OR = 0.6, 95%CI: 0.4, 0.9) were more common in the ED.

Table 5 shows the results of a multilevel logistic regression comparing clinical problem, type of liaison intervention, and setting according to whether it was a first or a repeat contact. A ratio above 1 indicates that the referral was more likely to be a repeat contact rather than first contact. There were differences in both clinical problems and interventions.

**Table 5:** Multilevel logistic regression for initial or repeated contact

*Variables*	*OR*	*(95% CI)*	*p-value*
Intercept	1.0	(1.3–6.0)	0.99
Main clinical problem for contact			
Other (reference)	1.0		
Adjustment to illness	1.8	(1.0–3.2)	0.05
Medical unexplained symptoms	3.2	(1.5–6.6)	<.001
Psychiatric symptoms	1.7	(1.2–2.3)	<.001
Cognitive impairment	1.7	(1.1–2.5)	0.02
Self-harm	1.0	(0.7–1.5)	0.84
Acute behaviour disturbance	1.8	(1.1–2.9)	0.03
Alcohol and/or drugs	1.2	(0.8–1.9)	0.31
Type of intervention			
Other (reference)	1.0		
Assessment and diagnosis formulation	0.4	(0.3–0.5)	<.001
Providing advice/signposting	0.7	(0.5–0.9)	0.01
Management of risk	0.7	(0.5–0.9)	0.02
Assessment of mental capacity/MHA	1.6	(1.0–2.6)	0.08
Medication management	0.9	(0.6–1.4)	0.73
Management of disturbed behaviour	1.2	(0.7–2.1)	0.40
Location of referral			
Other (reference)	1.0		
ED	0.4	(0.3–0.7)	<.001
Ward	1.6	(1.0–2.3)	0.03

*95% CI* 95% confidence interval

Repeat contacts were more likely than initial contacts to involve patients with psychological adjustment to physical illness (OR = 1.8, 95% CI: 1.0, 3.2), medically unexplained symptoms (OR = 3.2, 95% CI: 1.5, 6.6), psychiatric symptoms (OR = 1.7, 95% CI: 1.2, 2.3), cognitive impairment (OR = 1.7, 95% CI: 1.1, 2.5), and acute behaviour disturbance (OR = 1.8, 95% CI: 1.1, 2.9). Repeat contacts were less likely to involve the following interventions: assessment and diagnosis formulation (OR = 0.4, 95% CI: 0.3, 0.5), providing advice/signposting (OR = 0.7, 95% CI: 0.5, 0.9) and management of risk (OR = 0.7, 95% CI: 0.5, 0.9).

Initial contacts were more common in the ED (OR = 0.4, 95% CI: 0.3, 0.7) and repeat contacts in ward settings (OR = 1.6, 95% C I:1.0, 2.3).

### Differences between the different models of LP services

There were no significant differences between the four models of liaison service identified by our previous research in terms of location of referrals (i.e. ward or ED) or any of the reasons for referral or type of intervention delivered by the liaison services. There was great variation between hospitals, even within those who were delivering a similar liaison model, in terms of types of referral and forms of intervention. Supplementary Table 1 shows the median and interquartile ranges for location of referral, type of referral and liaison intervention.

## Discussion

This is the largest, most comprehensive survey of liaison activity in England that has been carried out to date, involving 18 different hospitals. Our findings suggest that on average liaison services see approximately 85 patients per week, but there are unsurprisingly large variations between individual services, and this figure should not be regarded as a standard benchmark for all liaison services. We estimated that activity corresponded to an average of 3% of ward admissions, which is higher than that reported by the ECLW study (1.4%) but in the mid-range of rates reported by other studies (0.5–5%) [4–9]. Four per cent represents a small fraction of the 25% of adults of working age and the 60% of older people in the general hospital who have mental health problems [1, 36].

The mean consultation rate in ED was 1.6%, where 4% of attendees are estimated to have mental health problems, suggesting better coverage in ED than the wards, but still less than half of the people who attend ED on average with a mental health problem have access to a mental health specialist.

Liaison activity is also highly correlated with the number of ED attendances and hospital admissions of an acute hospital, but not the number of its beds. So liaison activity is related to the flow of patients through an acute hospital rather than its actual size. Currently the recommended staffing levels of liaison services are based on the size of the hospital in terms of beds [37], and these recommendations may need to be modified based upon our findings.

There is an expectation that the expansion of liaison psychiatry services across most hospitals in England will lead to cost savings primarily through reducing in-patient length of stay, particularly for elderly patients with delirium/dementia [13]. Such an impact however is unlikely to result in a large effect if most LP services only assess and treat such a small percentage of patients with mental health problems in the acute hospital setting.

As in the ECLW study [3], co-morbid psychiatric symptoms and self -harm remain the two most common clinical scenarios that liaison teams manage, but cognitive disturbance has replaced medically unexplained symptoms as the third most common mental problem seen by liaison services in England. One quarter of liaison work, however, involves managing people with a range of other clinical problems, including e.g. drug and alcohol problems, medically unexplained symptoms, or psychological adjustment to illness. Liaison staff therefore require a wide range of skills to manage such clinical diversity.

The majority of liaison work in England is carried out by liaison nurses, which reflects the larger number of nurses employed in LP services compared to psychiatrists. We found differences between doctors and nurses in their clinical roles, both in terms of the kinds of clinical problems they saw and the interventions they delivered. Doctors were more likely to see patients with clinical problems that required specific medical skills, including the assessment and management of psychiatric symptoms in the context of physical illness; acute behavioural disturbance in a medical setting, and medically unexplained symptoms-of sufficient severity to prompt attendance at ED or admission to an acute hospital bed. Doctors were more likely to provide interventions that required specific medical knowledge (medication management), specific psychiatric training (Mental Health Work) or knowledge of psychopharmacology, particularly its use in the medically ill.

Other clinical problem areas were shared equally between both professions, but nurses were more likely to provide sign-posting and advice than doctors, which may reflect their role in seeing less complex patients than doctors, and their greater presence in ED as opposed to ward work. Our findings support previous work suggesting that doctors are an essential component of a general hospital LP services, with their input being particularly required in ward- based liaison work [38] due to the complex interplay between physical and mental health problems in patients who present to liaison services [16]. A recent systematic review also found that medical input also improves quality of care in ED settings [18] . There are no clear clinical criteria for determining which patients should be first assessed by a mental health nurse or a psychiatrist. The results of this study may provide some guidance for clinical teams regarding referral allocation.

We found considerable differences in the kind of clinical work that LP teams undertake in ED and ward settings. ED work was much more likely to involve assessment, diagnosis and risk management with patients presenting with drug and alcohol problems or self-harm. Comparatively, ward work was much more likely to involve dealing with patients with co-morbid physical and mental health problems, including psychological adjustment to physical illness, medication management and interventions for people with medically unexplained symptoms. Becker and colleagues also found that medication management was more common in a ward setting [21], and a previous publication has also described the complexity of ward work and the challenging nature of delivering mental health care in the general hospital setting [16]. The distribution of the different interventions in that previous publication [16] were very similar to that in the current study (e.g. assessment 32.6% [16] versus 34.6% present study; risk assessment 16.3% [16] versus 15.3 current study; medication management 16.6% [16] versus 10.1% current study and behavioural disturbance 8.1% [16} versus 8.1% current study.

Only a small number of services reported using any form of a standardised outcome measure, including those recommended by FROM-LP. Two possible explanations for the lack of the use of standardised outcome measure may be, the lack of a LP specific outcome measure and the difficulty that patients have in completing self-report measures in an acute setting [39]. Previously, a balanced scorecard approach has been suggested rather than generic outcome measures – for example an alcohol measure for alcohol problems, a cognitive measure for cognitive problems - due to the wide variety of different problems seen by liaison teams [39]. However this is often impractical as teams may not have the relevant measures to hand, and the scores from different outcome measures cannot be aggregated to obtain an overall indicator of outcome. Thus, generic measures are difficult to use with all patients especially in self-report format and yet condition-specific measures are impractical. This dilemma is important to resolve given that the recent investment in liaison services in England comes with an expectation that services will improve clinical reporting. The development of a new liaison-specific clinician-rated measure, may help to address this significant problem area [40].

Recent discussion of liaison services has emphasised their function as a rapid assessment and sign posting service [13, 37]. However nearly half the contacts in our survey were follow-up contacts, particularly in the ward setting. Our findings suggest that a substantial part of liaison work involves at least two contacts and is more interventional than a simple assessment and signposting service, mirroring the complexity and specialist nature of the work. The degree of in-hospital follow up will need to be factored into estimates of activity by commissioners when planning services, as activity is currently based on numbers of referrals to liaison services. A recent study from Bristol reported an average of 106 new referrals per month for the period October 2016 to September 2017 which would equate to 25 new referrals per week [24]. However, the results of this present study suggest that work load, in terms of face to face patients contact, of a typical service is at least double that.

Our survey focused on actual face-to-face contacts carried out by LP services during the survey period and did not collect data on other forms of contact, or other work involving the liaison teams. For example, it has been estimated that for each face to face liaison contact lasting one hour, there is a minimum of two additional hours of documentation work, to ensure an accurate clinical record and risk assessment has been recorded on the hospital’s / Trust’s electronic system(s) [41]. Other work of LP services include discussions with other clinical staff caring for the patient, social workers, family and carers and GPs. Additionally, educational work involving acute hospital staff, training and professional development, telephone advice, administrative work, audit, team meetings, case presentations, case management meetings, and management responsibilities add to the LP service work load. Many services also undertake a considerable amount of service development work. The activity figures therefore provide a proxy for the overall workload of teams.

We found that only 3% of all patients seen by LP services were referred to the Improving Access to Psychotherapy Treatment (IAPT) services, which in England, has been charged by government to provide treatment for patients with medically unexplained symptoms and depression in long term conditions. A recent study which examined the quality of care of older patients with depression in a general hospital setting found no patients were referred to IAPT or an equivalent psychological treatment service [42]. This suggests there is a serious disconnect between government policy and delivery of treatment. Considerable resources have been allocated to IAPT, which has become the main provider of psychological treatment services in most areas of England, so it is unclear why so few patients are referred to IAPT.

There are several limitations in relation to this study that require consideration.

First, although the liaison services that agreed to participate in this study were distributed across England, and included services according to each of our four types, it is possible that they differed in some respects to those that declined or were unable to participate. One potential bias could be that services that declined or were unable to participate may have perceived themselves as too busy to participate, which may result in an underestimate of activity.

Second, the activity of a liaison service can vary from week to week, and month to month. Therefore, LP service activity over 1 week may not reflect its annual throughput.

Third, our estimate of service activity as a percentage of ward admissions or ED attendances should be treated with caution, due to the variability in referral rates throughout the year to LP services, and the variability in hospital admissions and ED attendances.

Fourth, services were able to choose the week they carried out the survey, which may have affected activity levels, although as nearly all services were demand led, and have to respond to referrals within strict time constraints, it is unlikely to have had a major impact.

Fifth, we did not assess the quality of the work undertaken, so could not apply any quality standards such as those used by the Royal College of Psychiatrists [43].

Sixth, we were unable to determine the intervention frequency as the study was basically a snap shot of activity during a 1 week period. Previous work has suggested that approximately half of patients in a ward setting are seen on one occasion, with one quarter receiving 2–4 contacts, one fifth with 5–10 contacts and a small proportion requiring more than 10 contacts [16].

Finally, returns were heavily oriented towards the acute component of liaison work, and although we noted that nearly 10% of referrals were followed up in specialist liaison services we captured very little of the highly specialised work we know happens in about a quarter of LP services - for example specialist outpatient clinics and links to transplant, renal or burns units and other regional or sub-regional services. A more comprehensive survey of LP service provision would include this diverse low-volume high-intensity work.

## Conclusions

Liaison psychiatry services in England currently see a small proportion of patients in the general hospital setting with both physical and mental health problems. There is great variation between services in terms of activity. Liaison mental health nurses carry out most of the consultations, especially in the Emergency Department. Liaison mental health nurses and doctors perform different roles within liaison mental health services, with doctors undertaking more complex work.

## Supplementary information

**Additional file 1 Supplementary Table 1**. Main reason for referral and type of liaison intervention according to the four different types of liaison service.

## Data Availability

Data from this study are available by request from AH subject to appropriate terms and conditions.

## Abbreviations

LP Services: Liaison Psychiatry Services
ED: Emergency Department
IAPT: Improving Access to Psychological Treatment services
CI: Confidence Interval
GPs: General Practitioners
FROM-LP: Framework for Routine Measurement in Liaison Psychiatry
IRAC: Identify and Rate the Aim of the Contact
OR: Odds ratio
HES: Hospital Episode Statistics

## References

[1] Department of Health. No Health Without Mental Health: A Cross-Government Mental Health Outcomes Strategy for People of all Ages. London; 2011.

[2] Barratt H, Rojas-Garcia A, Clarke K, Moore A, Whittington C, Stockton S, Thomas J, Pilling S, Raine R (2016). Epidemiology of mental health attendances at emergency departments: systematic review and meta-analysis. PLoS One.

[3] Huyse FJ, Herzog T, Lobo A, Malt UF, Opmeer BC, Stein B, Cardoso G, Creed F, Crespo MD, Guimaraes-Lopes R (2000). European consultation-liaison services and their user populations: the European consultation-liaison workgroup collaborative study. Psychosomatics.

[4] Diefenbacher A (2001). Implementation of a psychiatric consultation service: a single-site observational study over a 1-year-period. Psychosomatics.

[5] Christodoulou C, Fineti K, Douzenis A, Moussas G, Michopoulos I, Lykouras L (2008). Transfers to psychiatry through the consultation-liaison psychiatry service: 11 years of experience. Ann General Psychiatry.

[6] Su JA, Chou SY, Chang CJ, Weng HH (2010). Changes in consultation-liaison psychiatry in the first five years of opearation of a newly-opened hospital. Chang Gung Med J.

[7] Sanchez-Gonzalez R, Rodriguez-Urrutia A, Monteagudo-Gimeno E, Vieta E, Perez-Sola V, Herranz-Villanueva S, Pintor-Perez L (2018). Clinical features of a sample of inpatients with adjustment disorder referred to a consultation-liaison psychiatry service over 10years. Gen Hosp Psychiatry.

[8] Sanchez-Gonzalez R, Bailles-Lazaro E, Bastidas-Salvado A, Lligona A, Herranz-Villanueva S, Perez-Sola V, Pintor-Perez L (2018). Clinical profile of inpatients referred to a consultation-liaison psychiatry service: an observational study assessing changes over a 10-year period. Actas Esp Psiquiatr.

[9] Gala C, Rigatelli M, De Bertolini C, Rupolo G, Gabrielli F, Grassi L (1999). A multicenter investigation of consultation-liaison psychiatry in Italy. Italian C-L Group. Gen Hosp Psychiatry.

[10] NHS England. Five year forward view. London; 2014. p. 1–39.

[11] NHS England. The long term plan. London; 2019. p. 1–134.

[12] Wood R, Wand APF (2014). The effectiveness of consultation-liaison psychiatry in the general hospital setting: a systematic review. J Psychosom Res.

[13] Tadros GSR, Kingston P, Mustafa N, Johnson E, Pannell R, Hashmi M (2013). Impact of an integrated rapid response psychiatric liaison team on quality improvement and cost savings: the Birmingham RAID model. Psychiatrist.

[14] Andreoli PB, Citero Vde A, Mari Jde J (2003). A systematic review of studies of the cost-effectiveness of mental health consultation-liaison interventions in general hospitals. Psychosomatics.

[15] Callaghan P, Eales S, Coates T, Bowers L (2003). A review of research on the structure, process and outcome of liaison mental health services. J Psychiatr Ment Health Nurs.

[16] Guthrie E, McMeekin A, Thomasson R, Khan S, Makin S, Shaw B, Longson D (2016). Opening the 'black box': liaison psychiatry services and what they actually do. BJPsych Bull.

[17] Mujic F, Cairns R, Mak V, Squire C, Wells A, Al-Harrasi A, Prince M (2018). Liaison psychiatry for older adults in the general hospital: service activity, development and outcomes. BJPsych Bull.

[18] Evans RCJ, Ablard S, Rimmer M, O'Keeffe C, Mason S (2019). The impact of different liaison psychiatry models on the emergency department: a systematic review of the international evidence. J Psychosom Res.

[19] Opmeer B (2017). Extending liaison psychiatry service improves care and reduces costs for self-harm patients. Br J Hosp Med.

[20] Kaner EPK, Vale L, McGovern R, Stamp E, Albani V, O’Donnell A, Crowe L (2013). Liaison psychiatry service evaluation. NHS Sunderland commissioning group. Newcastle University. Newcastle University: NHS Sunderland Commissioning Group.

[21] SR BL, Hardy R, Pilling S (2016). The Raid model of liaison psychiatry: report on the evaluation of four pilot Services in East London.

[22] Walker ABJ, Lee W, Guthrie E, West R, Trigwell P, Quirk A, Crawford M, House A (2018). The Organisation and Delivery of Liaison Psychiatry Services in General Hospitals in England: results of a National Survey. BMJ Open.

[23] Trigwell P, Kustow J (2016). A multidimensional framework for routine outcome measurement in liaison psychiatry (FROM-LP). BJPsych Bull.

[24] Guest C, Crockett S, Little P, Patel A (2018). The clinical utility of the IRAC component of the framework for routine outcome measurement in liaison psychiatry (FROM-LP). BJPsych Bulletin.

[25] Vandenbroucke JP, von Elm E, Altman DG, Gotzsche PC, Mulrow CD, Pocock SJ, Poole C, Schlesselman JJ, Egger M, Initiative S (2007). Strengthening the reporting of observational studies in epidemiology (STROBE): explanation and elaboration. Epidemiology.

[26] NHS Digital (2018). Emergency care data set.

[27] Finch WHBJ, Kelley K (2014). Multilevel Modelling Using R. : Taylor & Francis Group.

[28] Aaparouhov T (2006). General multi-level modeling with sampling weights***.*** Communications in Statistics. Theory Methods.

[29] Carle AC (2009). Fitting multilevel models in complex survey data with design weights: recommendations. BMC Med Res Methodol.

[30] Hsieh FY, Lavori PW, Cohen HJ, Feussner JR (2003). An overview of variance inflation factors for sample-size calculation. Eval Health Prof.

[31] Guy W (1976). ECDEU Assessment Manual for Psychopharmacology. USA: US Department of Heath,Education, and Welfare Public Health Service Alcohol, Drug Abuse, and Mental Health Administration.

[32] Pangman VC, Sloan J, Guse L (2000). An examination of psychometric properties of the mini-mental state examination and the standardized mini-mental state examination: implications for clinical practice. Appl Nurs Res.

[33] Zigmond AS, Snaith RP (1983). The hospital anxiety and depression scale. Acta Psychiatr Scand.

[34] Wing J, Curtis RH, Beevor A (1999). Health of the Nation Outcome Scales (HoNOS). Glossary for HoNOS score sheet. Br J Psychiatry.

[35] Evans C, Connell J, Barkham M, Margison F, McGrath G, Mellor-Clark J, Audin K (2002). Towards a standardised brief outcome measure: psychometric properties and utility of the CORE-OM. Br J Psychiatry.

[36] Creed F, Morgan R, Fiddler M, Marshall S, Guthrie E, House A (2002). Depression and anxiety impair health-related quality of life and are associated with increased costs in general medical inpatients. Psychosomatics.

[37] NHS England. Guidance for commissioning liaison psychiatry services. London; 2014.

[38] Guthrie EA, McMeekin AT, Khan S, Makin S, Shaw B, Longson D (2017). An analysis of whether a working-age ward-based liaison psychiatry service requires the input of a liaison psychiatrist. BJPsych Bull.

[39] Fossey M, Parsonage M (2012). Outcomes and Performance in Liaison9Psychiatry.Developing a Measuring Framework.

[40] Guthrie E, Harrison M, Brown R, Trigwell P, Abraham S, Kelsall P, Sandhu R, Thomasson R, Nawaz S (2017). The development of an outcome measure for consultation liaison psychiatry. J Psychosom Res.

[41] Jasmin K, Walker A, Guthrie E, Trigwell P, Quirk A, Hewison J, Murray CC, House A (2019). Integrated liaison psychiatry services in England: a qualitative study of the views of liaison practitioners and acute hospital staffs from four distinctly different kinds of liaison service. BMC Health Serv Res.

[42] Shastri AAL, Tooke B, Quirk A, Corrado O, Hood C, Crawford MJ (2019). Recognition and treatment of depression in older adults admitted to acute hospitals in England. Clin Med.

[43] Brightey-Gibbons F, Patterson E, Rhodes E, Ryley A, Hodge S (2017). Royal Quality Standards for Liaison Psychiatry Services. Royal College of Psychiatrists Centre for Quality Improvement.

