# Supplementary material for: The nature and activity of liaison mental services in acute hospital settings: a multi-site cross sectional study
Source: BMC Health Serv Res. 2020 Apr 15;20:308. doi: 10.1186/s12913-020-05165-x (PMC7157982; doi:10.1186/s12913-020-05165-x)
Supplement: Supplementary file 1 — Additional file 1 Supplementary Table 1. Main reason for referral and type of liaison intervention according to the four different types of liaison service. [file 12913_2020_5165_MOESM1_ESM.docx]

Supplementary Table 1. Main reason for referral and type of liaison intervention according to the four different types of liaison service.

|  | ***Cluster 1*** | ***Cluster 2*** | ***Cluster 3*** | ***Cluster 4*** |
| --- | --- | --- | --- | --- |
|  | **Median IQR** | Median | Median IQR | Median IQR |
|  |  |  |  |  |
| **Main reason for referral** | |  |  |  |
| Adjustment to illness | 1.4 (0.2-6.9) | 2.3 | 6.5 (2.6-9.3) | 2.3 (1.7-3.3) |
| Medical unexplained symptoms | 1.8 (0.1-7.0) | 1.2 | 3.3 (2.8-4.3) | 1.5 (0.2-2.4) |
| Psychiatric symptoms | 28.3 (1.8-43.0) | 35.4 | 33.4 (28.5-44.2) | 34.2 (26.2-39.1) |
| Cognitive impairment | 8.6 (0.6-30.8) | 27.1 | 16.3 (10.1-31.0) | 9.3 (3.0-14.7) |
| Self-harm | 35.8 (10.6-46.2) | 13.8 | 15.8 (12.9-29.1) | 32.0 (21.8-44.9) |
| Acute behaviour disturbance | 8.3 (3.5-10.3) | 10.2 | 7.1 (2.7-9.0) | 4.7 (4.0-5.7) |
| Alcohol and/or drugs | 6.9 (3.5-7.1) | 8.9 | 5.4 (4.4-7.5) | 11.9 (8.8-17.6) |
| Other | 0.1 (0.1-0.2) | 1.2 | 2.7 (1.2-5.2) | 3.0 (0.0-7.7) |
|  |  |  |  |  |
| **Type of liaison intervention** |  |  |  |  |
| Assessment and diagnosis formulation | 32.6 (18.3-48.0) | 34.0 | 33.8 (27.0-37.0) | 30.9 (24.5-43.8) |
| Providing advice/signposting | 27.9 (16.9-35.0) | 23.2 | 16.3 (14.0-26.9) | 23.2 (8.9-31.6) |
| Management of risk | 18.1 (0.9-30.9) | 18.1 | 21.0 (12.5-28.6) | 27.8 (22.8-36.3) |
| Assessment of mental capacity/MHA | 2.8 (1.8-4.6) | 5.2 | 5.4 (2.8-7.5) | 3.3 (1.1-7.7) |
| Medication management | 6.9 (4.1-12.4) | 6.9 | 8.5 (4.2-15.8) | 2.1 (0.2-4.4) |
| Management of disturbed behaviour | 2.1 (0.1-5.6) | 10.8 | 5.2 (3.2-11.3) | 4.2 (1.5-12.5) |
| Other | 2.7 (0.2-5.6) | 1.8 | 4.3 (1.7-10.4) | 3.9 (1.3-8.0) |

IQR = interquartile range; cluster 2 has only two hospitals, no IQR reported
